# Prenatal diagnosis of a novel 7q31.31q31.33 microduplication with a favorable outcome

**DOI:** 10.1186/s13039-022-00589-y

**Published:** 2022-03-26

**Authors:** Huili Luo, Linlin Liu, Yuexiang Feng

**Affiliations:** 1 Medical Laboratory Technology, Shiyan Maternal and Child Health Hospital, Shiyan, Hubei People’s Republic of China; 2 Hubei Provincial Center for Disease Control and Prevention, Institute of Health Inspection and Testing, Wuhan, Hubei People’s Republic of China; 3 Department of Endocrinology, Wuhan Hankou Hospital, Wuhan, Hubei People’s Republic of China

**Keywords:** Copy number variants, Chromosomal microarray analysis, Prenatal diagnosis, Unbalanced chromosome abnormality (UBCA)

## Abstract

**Background:**

Copy number variants (CNVs) are an important source of normal and pathogenic genome variations. Especially CNVs identified in prenatal cases need careful considerations and correct interpretation if those are harmless or harmful variants from the norm.

**Case presentation:**

Herein, we reported a paternally inherited duplication of 7.6 Mb in 7q31.3 with, surprisingly, a favorable outcome. GTG-banding and CMA on the DNA derived from uncultured amniocytes revealed a karyotype: 46,XX.arr[GRCh37] 7q31.31q31.33(118,601,001_126,177,044) × 3. Ultrasound examination showed no dysmorphisms or intrauterine growth restriction in the fetus and the father was clinically normal as well.

**Conclusion:**

Prenatal detection of a 7.6 Mb in 7q31.31 to 7q31.33 duplication in a female fetus turned out to be a yet unreported unbalanced chromosome abnormality. This is another example that parental testing and GTG-banding are necessary additional tests to be done in prenatal cases, before a reliable conclusion on the meaning of an aberration can be drawn.

## Background

Copy number variants (CNVs) are an important source of normal and pathogenic genome variations [1]. While many CNVs are known since almost 2 decades for European population, recent widespread application of chromosomal microarray analysis (CMA) in diagnosis in China revealed many new CNVs. Overall, clinical significance of these new CNVs needs to be still some considerations [2, 3].

Talking about prenatally detected CNVs in the range of several mega base pairs (Mb) in size the great majority of those will lead to adverse effects for the carrier. However, rarely there are so-called unbalanced chromosome abnormalities (UBCAs), which, irrespective of their size, do not cause any, or less than expected harm to their carriers [4–7].

Herein, we reported a family with a 7.6 Mb duplication in7q31.3 with a favorable outcome, being a new example of a UBCA.

## Case report

**Fig. 1:**
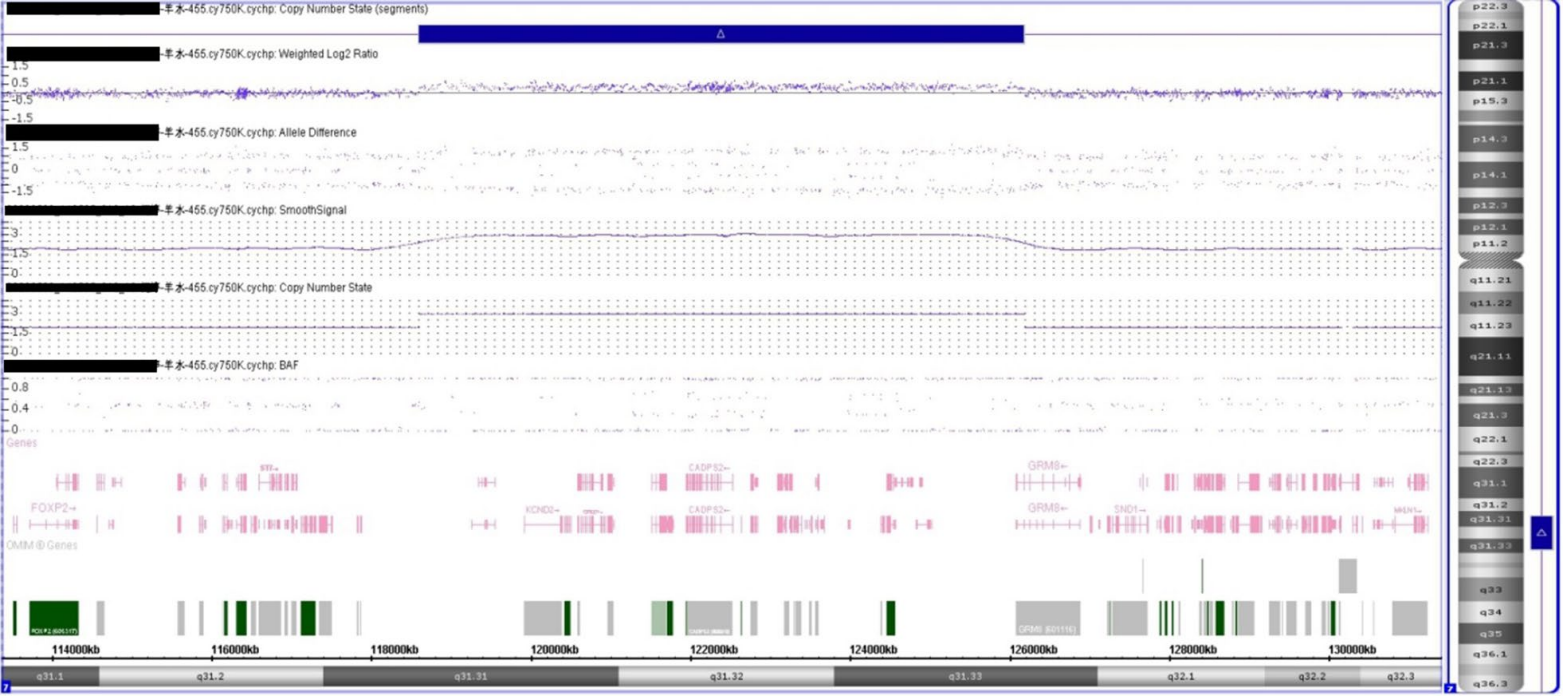
Depiction of the terminal 7.6-Mb duplication revealed by CMA (blue marked region); the final result for the fetus was 46,XX.arr[GRCh37] 7q31.31q31.33(118,601,001_126,177,044) × 3

**Fig. 2:**
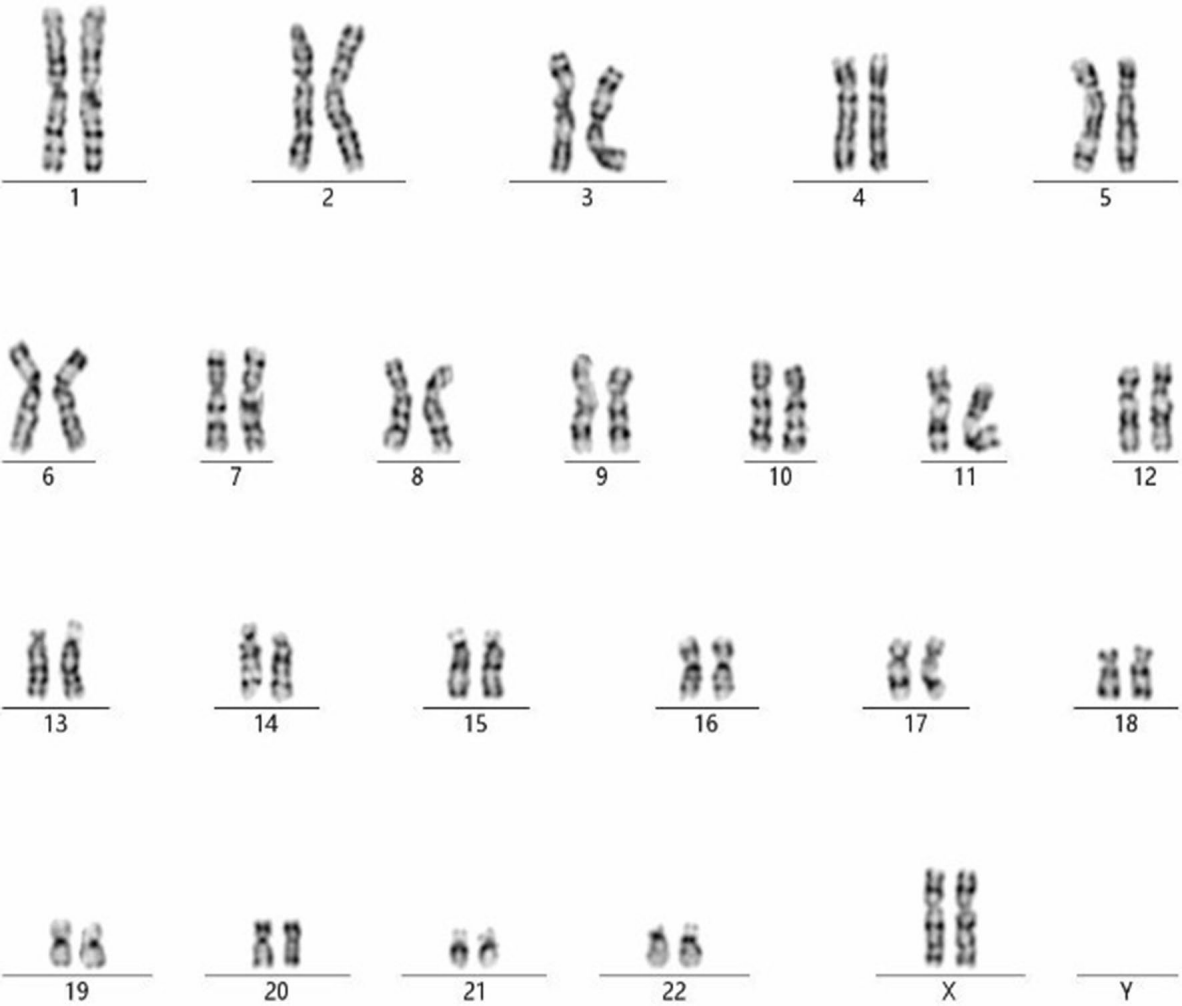
GTG-banding result of the fetus with the cryptic dup(7) (q31.31q31.33)

A 36-year-old gravida-1-para-0 pregnant women had amniocentesis at 18 weeks of gestation, due to advanced maternal age. She and her 39 years old husband reported no family history of birth defects or genetic diseases. CMA by Affymetrix CytoScan 750 K chip, which includes 550 k non-polymorphic markers and 200 k SNP markers performed on DNA derived from uncultured amniocytes identified a 7.6 Mb chromosomal duplication (Fig. 1), while GTG-banding of fetus and parents were normal (Fig. 2). The karyotype for the fetus was according to International System of Cytogenomic Nomenclature 2020 (ISCN 2020) [8] 46,XX.arr[GRCh37] 7q31.31q31.33(118,601,001_126,177,044) × 3. Parental CMA showed that the father had a duplication of the same region as the fetus.

Sonography revealed no hints on dysmorphisms or intrauterine growth restriction (IUGR) in the fetus, and a comprehensive physical examination of the parents, especially the father showed no abnormalities. After genetic counseling, the parents decided to continue the pregnancy. At 38 weeks of gestation, a female baby weighing 3,300 g was delivered vaginally. The baby received a complete physical examination, and the results were normal. At the 24-month checkup, the baby was developing normally.

## Discussion

According to the literature [6] yet four UBCA regions with duplications are known along chromosome 7: 7pter to 7p22.3, 7p13 to 7p12.2, 7p11.2 to 7q11.22 and 7q32 to 7q36.1. This study adds one more UBCA region as 7q31.31 to 7q31.33.

While in many cases families with UBCA have a degree of phenotypic effects [6], the here reported family belongs to the smaller group of UBCA cases without any clinical signs or symptoms. Also the size of almost 8 Mb is unusually large even for UBCAs [6]. As being also typical for UBCAs there are also reports on similar duplications which show—in parts severe clinical consequences [9–11], like facial dysmorphism, moderate intellectual disability, autistic spectrum disorder, and epilepsy [11].

In the present case the duplicated region contains multiple genes: *KCND2*, *TSPAN12*, *ING3*, *WNT16*, *FAM3C*, *PTPRZ1*, *AASS*, *FEZF1*, *CADPS2*, *TAS2R16*, *SLC13A1*, *NDUFA5*, *LMOD2*, *WASL*, *HYAL4*, *SPAM1*, *GPR37*, *POT1*, and *GRM8*. Among these genes, *CADPS2*, *WNT16*, and GRM8 *play* essential roles in autism spectrum disorders. Besides, *TSPAN12*, *ING3*, *FAM3C*, *PTPRZ1*, *FEZF1*, *WASL*, *HYAL4*, *GPR37*, and *POT1* are cancer-related genes [12, 13]. Still, yet no triplosensitivity genes in this region have been identified. The latter may be the reason for symptom freeness of the father and his daughter in the reported case.

## Conclusion

Herein the first case of a (sub)chromosomal imbalance expressed as duplication in 7q31.31 to 7q31.33 is presented, which is per definition an UBCA without obvious clinical consequences for two carriers within the same family. The case highlights that prenatal detection of even large CNVs implicates parental testing to come to a well-funded estimation on the impact of the identified alteration.

## Data Availability

Please contact the corresponding author for data requests.

## Abbreviations

CNVs: Copy number variants
CMA: Chromosomal microarray analysis
UBCA: Unbalanced chromosome abnormality
